# Our Relationship to Water and Experience of Water Insecurity among Apsáalooke (Crow Indian) People, Montana

**DOI:** 10.3390/ijerph18020582

**Published:** 2021-01-12

**Authors:** Christine Martin, Vanessa W. Simonds, Sara L. Young, John Doyle, Myra Lefthand, Margaret J. Eggers

**Affiliations:** 1 Crow Tribe of Indians, Crow Agency, MT 59022, USA; saralyoung@hotmail.com (S.L.Y.); doylej@lbhc.edu (J.D.); myrajlefthand@gmail.com (M.L.); 2 Crow Water Quality Project, Little Big Horn College, Crow Agency, MT 59022, USA; 3 Crow Environmental Health Steering Committee, Little Big Horn College, Crow Agency, MT 59022, USA; mari.eggers@montana.edu; 4 Department of Health and Human Development, Montana State University, Bozeman, MT 59717, USA; 5 Center for Health Equity Research, Northern Arizona University, Flagstaff, AZ 86001, USA; 6 National Environmental Justice Advisory Council, Environmental Protection Agency, Washington, DC 20460, USA; 7 Department of Microbiology & Immunology, Montana State University, Bozeman, MT 59717, USA

**Keywords:** Native Americans, water insecurity, environmental justice, health disparities

## Abstract

Affordable access to safe drinking water is essential to community health, yet there is limited understanding of water insecurity among Native Americans. Therefore, the focus of this paper is to describe Apsáalooke (Crow Indian) tribal members’ experiences with water insecurity. For Apsáalooke people, local rivers and springs are still vitally important for traditional cultural activities. We interviewed 30 Native American adults living on the Crow Reservation in Southeastern Montana. Participants answered six open-ended interview questions about their water access, costs of obtaining water and changes in their domestic and traditional water uses. Participants emphasized how the use of water has changed over time and described the complex challenges associated with addressing water insecurity in their community, including the importance of considering the spiritual and cultural impacts of water insecurity on health. Water insecurity is a growing global problem and more attention and efforts are needed to find appropriate and affordable solutions.

## 1. Introduction

Affordable access to safe drinking water is essential to community health [1,2,3], yet research to understand water insecurity and effective interventions in the United States (US) is still an emerging field [4]. Multiple definitions of “water insecurity” exist, ranging from the utilitarian, “inadequate or inequitable access to clean, safe and affordable water for drinking, cooking, and sanitation and hygiene” [4] to broader, but still material definitions that encompass human and ecosystem health, sustainability, productive capacity, and more. This material approach to defining water security is exemplified by the United Nation’s Sustainable Development Goal #6 (UN SDG 6): “Ensure access to water and sanitation for all” [5]. While the UN’s SDGs may commonly be understood as applicable to international development and improvement of human wellbeing in lower and middle income countries, they are also relevant in minority and other underserved communities in the United States (US) [6,7,8] Indeed, tools from international development, such as the Water Poverty Index (WPI) [9], have been employed to assess water justice issues in *colonias* in the US [10]. The WPI, developed with the participation of diverse stakeholders in Sri Lanka, Tanzania and South Africa, combines material “measures of […] access to water; water quantity, quality and variability; water uses (domestic, food, productive purposes); capacity for water management; and environmental aspects” [9]. Progress continues on creating multidimensional and translatable water insecurity metrics which can be employed across low, middle, and high income countries (e.g., [6,7,8,9,10]).

A current, systematic literature review of water insecurity in the US finds that a variety of community-engaged, culturally relevant policy and intervention strategies are being piloted, from implementing health education and improving affordability of public water service to mapping water scarcity and safe drinking water violations [4]. These authors recommend four key strategies as a basis for reducing water insecurity disparities: (a) Assessment of communities impacted by water scarcity, (b) identification of water scarce regions, (c) recognition of the human right to water, and (d) the elucidation of how communities experience water insecurity [4]. To address this last recommendation, these authors suggest that household level needs and experiences of water insecurity be assessed to “understand the impacts water insecurity has on daily life, health perceptions, and psychosocial well-being,” and to provide qualitative data complementary to more commonly used quantitative measures [4]. 

While this excellent review identifies various demographics particularly impacted by water insecurity, including the homeless and rural residents relying on home wells, it does not thoroughly address the literature on how low income and minority populations across the US are affected [11,12,13,14,15]. These communities are particularly vulnerable to inadequate safe water, as they are already challenged by other health, environmental, and economic disparities [16]. Lack of affordable access to safe water can exacerbate such existing disparities and undermine communities’ future prosperity [16]. 

For Native American communities, disparities in access to safe drinking water are among the most serious environmental public health challenges [2,16,17]. Throughout Indian Country, 13% of homes assessed in 2007 by the Indian Health Service, “lacked access to safe drinking water and/or safe wastewater disposal infrastructure […] compared to 0.6% of non-Native homes” assessed nationwide [17]. This disparity produces inequities in exposure to both microbial and chemical waterborne contaminants [11]. Further, rural families relying on home wells are typically counted in such statistics as having access to “safe” water, without any data on well water quality. However, it is well documented that private wells in the US are more likely than municipal supplies to have contaminants exceeding health standards [2,18,19]. In a nationwide US Geological Survey (USGS) study, more than 23% of home wells were deemed unsafe due to metals and/or nitrate, and 40%+ tested positive for coliform bacteria [19]. (While coliform bacteria in well water is not a confirmation of fecal contamination, its presence in groundwater does indicate risk of fecal contamination). As is true of many rural communities across the United States [19], substantial percentages of reservation residents rely on home wells [20]. Multiple studies have documented home wells contaminated by uranium and arsenic across the Navajo nation [21,22], and 39% of Tribal families’ wells on the Crow Reservation are unsafe for long term consumption due to metals and/or nitrate [23]. 

Climate change is exacerbating these existing challenges. A 2013 review of climate change effects on Tribal water resources addressed impacts to water supply, water infrastructure, culturally important aquatic species, ranching, agriculture, water rights, and soil quality [24]. While these authors acknowledge the spiritual importance of water, they say very little about the *direct* impacts of water quality and quantity deterioration on cultural practices and spiritual wellbeing, and hence on overall health [24]. 

Seeking to define Tribal water insecurity and understand its broad impacts on community wellbeing can benefit from the parallel literature on Tribal food insecurity in the United States. The Coast Salish Swinomish Indian Tribal Community in Washington State writes eloquently about impacts of declining access to their traditional foods. With environmental pollutants and ecosystem deterioration contaminating and reducing the availability of subsistence foods, not only is their nutrition being impacted, but social relationships, family economics, cultural practices, spiritual traditions, and emotional health are also being negatively affected [25]. For the Swinomish, local seafood is not only central to their diet but integral to their way of life. Environmental contamination is poisoning subsistence species they depend upon, and hence federal guidelines recommend they reduce their consumption. Some tribal members choose to “poison their body to nourish their soul,” explaining ([25], p. 103):

*…food security, ceremonial use, knowledge transmission, and community cohesion all play primary roles in Swinomish definitions of individual and community health and complement physical indicators of health. Thus, to eat less seafood (as prescribed on the basis of current physiological measures) may actually be detrimental to the Swinomish concept of health…*

Recent literature on water insecurity in Canadian Indigenous and Alaska Native communities is also directly relevant. An Inuit community in Labrador works with Hanrahan et al. [26] on documenting and mitigating local water insecurity issues, and this team has linked consumption of contaminated water with gastrointestinal disease, mental stress and poverty as well as chronic injuries from water hauling. A case study with a First Nations community in Yukon, Canada, examined how vital traditional water sources are “for meeting important health requirements including physical, spiritual, and cultural wellbeing” [27]. These authors argue for a more holistic approach to understanding water insecurity, recognizing historical and political root causes, and maintaining distinct Western and Indigenous ways of understanding water [27]. Members of multiple Indigenous communities in Saskatchewan, Canada similarly voice that “water security from an Indigenous perspective embraces much more than the material value of water,” and includes cultural, spiritual, and ethical aspects [28]. Mitchell [29] provides a recent review of water insecurity and Native American health, similarly concluding that “for Indigenous people, water takes on a much greater significance as it is connected to the culture, identity and livelihoods of Indigenous communities”. A recent publication describing the Hopi’s struggle to protect a critical sacred spring is one example of depicting direct cultural impacts [30]. 

The experiences of these Indigenous communities resonate with us as Apsáalooke (Crow) tribal members (and one non-Crow author who married into the Tribe). We began a grassroots effort in 2005 to research and understand Reservation-wide water contamination and related health issues [23,31,32,33,34,35,36,37,38,39]. Our research to identify and quantify contaminant levels and assess cumulative health risks has been tremendously helpful in community education and mitigation efforts. However, while we have quantitatively “assess[ed] […] communities impacted by water scarcity” (as recommended by [4]), we realized the impacts of water insecurity on community health and wellbeing were far broader than our quantitative methods captured. For our people, the changes in and deterioration and contamination of local ecosystems, including traditional water sources, are impacting not only our physical health, but also Tribal emotional and spiritual health, as environmental, tribal, and individual wellbeing are entirely interrelated [32,39,40,41]. The water insecurity created by water quality deterioration is impacting multiple aspects of community wellbeing, similar to the effects of subsistence food insecurities on the Swinomish community, and the impacts of water insecurity on Canadian and other Native nations. Water is sacred to us and therefore is critical for sustaining our communities’ health and wellbeing, allowing us to maintain who we are as a people [41].

Realizing that qualitative methods are essential to elucidating and sharing our Tribe’s experience of water insecurity, we began conducting interviews by and of our Elders in 2011. In undertaking this research, we sought to identify and illuminate Crow Tribal members’ experiences, needs, perceptions of, and strategies for coping with water insecurity as a result of contaminated traditional and current tribal water sources, including rivers, springs and home wells. 

## 2. Crow Reservation

**Figure 1:**
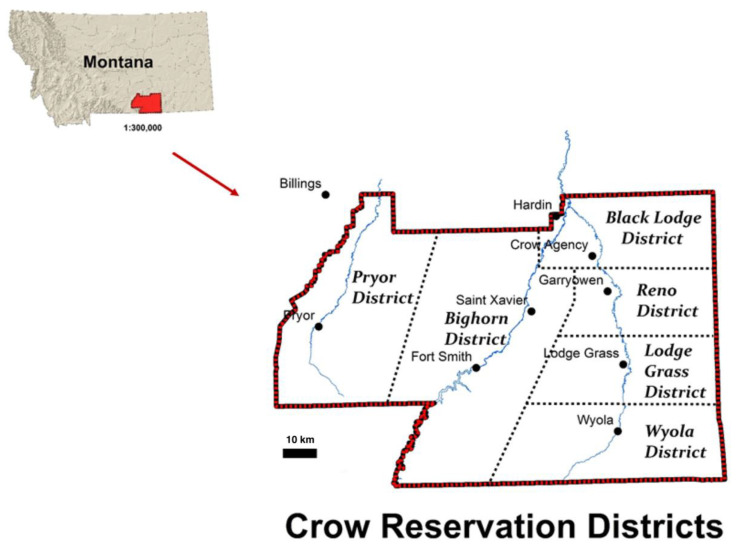
Map of the Crow Reservation, showing location within Montana, as well as major rivers, towns and districts of the Reservation [33]. All three rivers flow north, eventually joining the Yellowstone River.

The Crow Reservation, in Southcentral Montana, is home to nearly 70% of our Tribe’s 13,260 members [42]. The Reservation encompasses about 2.2 million acres of our Tribe’s traditional territory, including three mountain ranges and three large river valleys [43]. The majority of communities, including the “capital” town of Crow Agency, are situated in the Little Bighorn River valley, with smaller communities on the other rivers and creeks (Figure 1). The tribe was originally called “Apsáalooke,” meaning “children of the large-beaked bird” which was misinterpreted by white settlers as “Crow”. The Crow language is still widely spoken by people over 30, with some families and a new immersion school continuing to pass the language on to younger generations [44]. Many cultural traditions, including our clan system, continue to be practiced. (The authors do not wish to explain Apsáalooke religious traditions in this article. Readers interested in learning more are referred to [45,46,47,48].) Water has always been held in high respect among Tribal members, and river and spring waters are still used in many ceremonies [41]. Tribal Elders say they “grew up on these rivers,” spending their summers in the water and along the riverbanks, and still today children spend the hot summer days playing in and along the rivers. Older women remember going with their mothers and grandmothers to collect river water for domestic use, a practice that continued until indoor plumbing was installed in rural districts in the 1960s. Local riparian ecosystems are home to important medicinal plants, five species of berry shrubs, deer, and other species vital to food security and cultural identity. Additionally, water birch, sandbar willow, cottonwood, chokecherry, ash, and other riparian tree species continue to be collected for traditional practices and ceremonies. This country that has been our ancestral homeland for many centuries, hence our community—especially our older generation—retains significant traditional ecological knowledge.

The Reservation remains largely rural and agricultural. For the past 40+ years, our economy has been based primarily on income from the mining of our Tribe’s extensive coal reserves [42], although this is declining. Coal mining has been active in the very sparsely settled southeast corner of the Reservation, and elevated sodium and sodium absorption ratios have been found downstream of one mine [49]. However, the rivers in the mined watersheds drain southeast, off the Reservation, and hence the mining contamination impacts few Crow Tribal families. The Indian Health Service Hospital, the Bureau of Indian Affairs, Little Big Horn College (LBHC), local schools, service industries, and agriculture are other sources of employment [43]. Both Tribal and non-Tribal ranchers run small cattle operations and raise horses, especially in the Little Bighorn River Valley. Although there are feedlots as well as extensive dry land and irrigated agricultural operations, almost all of these are run by non-Tribal members. Water withdrawn from both the Bighorn and Little Bighorn Rivers for irrigation is managed by local water user associations, controlled by non-Native farmers and ranchers, and is distributed almost exclusively to these non-Tribal agricultural operations. In the Little Bighorn River valley, extensive withdrawals have lately been reducing the flow to ankle deep water in late summer, threatening the public water supply for the Tribe’s “capital” town of Crow Agency.

Agriculture expanded significantly beginning in the 1960s, and Elders remark that with those changes, the river water quality deteriorated. Families who had long been collecting river water daily for home use, say that the rivers used to “clear up” after spring runoff, but since the 1960s, the water has stayed murky all summer long. As kids spending summers along the rivers, they had played with local frogs, and have seen the populations of both frogs and river mollusks decline ever since [32]. With this evident deterioration of water quality, rural families switched from collecting river water to relying on their then newly installed home wells for domestic use. However, the groundwater across most of the Reservation has such high total dissolved solids, that the poor taste, “rotten” odor and discoloration of home well water are constant reminders of the loss of river water for domestic use [23]. 

Over time, our Reservation community became concerned that contaminated home well water was contributing to perceived cancer clusters. In 2005, a grassroots group of Crow Tribal members and an LBHC science faculty member decided to conduct a community-wide assessment of environmental health risks. Although we identified many potentially hazardous exposures, we came to a consensus that consumption of contaminated water presented the greatest environmental health risk to our Tribe. We subsequently formed the Crow Environmental Health Steering Committee (CEHSC) to understand, communicate, and mitigate these water-related health risks, and have been working together since 2005 [23,31,32,33,34,35,36,37,38,39,41]. Our Committee includes Tribal members from Elders (some with graduate degrees) to young adults, including college students. We collaborate with scientists at LBHC, Montana State University Bozeman and University of New Mexico, a local non-profit and a regional foundation, as well as with members of other Tribes across the country. 

**Figure 2:**
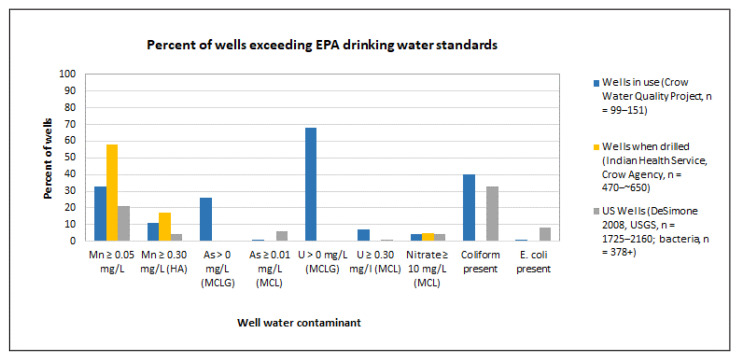
Percentages of home wells which exceed Environmental Protection Agency (EPA) drinking water standards for manganese (Mn), arsenic (As), uranium (U), nitrate, coliform, or *Escherichia coli,* comparing Crow Tribal home wells [23] with US Geological Survey (USGS) national data [19]. For manganese, 0.05 mg/L is the EPA secondary standard, and 0.30 mg/L is the Health Advisory. EPA MCLGs are Maximum Contaminant Level Goals and MCLs are Maximum Contaminant Levels for health. For nitrate, both the MCLG and the MCL are 10 mg/L [50]. The presence of coliform indicates risk of fecal contamination, while *E. coli* confirms it. “Wells when drilled” data courtesy of Indian Health Service Hospital, Crow Agency, MT. Absent values are “no data collected” [23].

**Figure 3:**
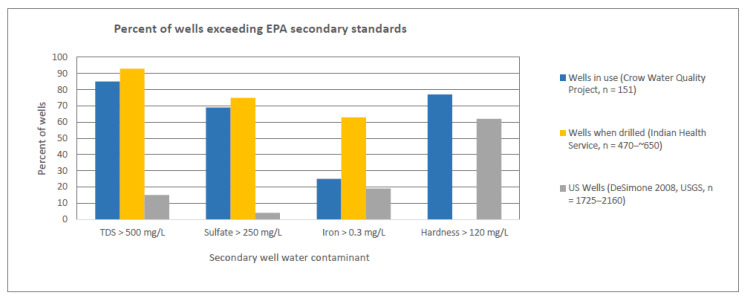
Percent of home wells exceeding EPA secondary drinking water standards for total dissolved solids (TDS), sulfate, iron and hardness [50], comparing Crow Tribal home wells with USGS national data [19,23]. Secondary Drinking Water Regulations provide “non-enforceable Federal guidelines regarding cosmetic effects (such as tooth or skin discoloration) or aesthetic effects (such as taste, odor, or color) of drinking water” [50]. Water exceeding 120 mg/L CaCO_3_ is considered “hard,” resulting in deposits in pipes and hot water heaters and scaling on fixtures, deteriorating plumbing. The Indian Health Service did not test for hardness.

Based on US Geological Survey data, about half of county residents rely on home wells for domestic water [19], including roughly 1020 Tribal families. Almost 40% of Tribal home wells tested (*n* = 164) were found to be unsafe for lifetime consumption due to the combination of manganese, uranium, arsenic, and/or nitrate and an overlapping 40%+ were coliform contaminated, indicating risk of fecal contamination (Figure 2). Combined, ~55% of homes have unsafe well water [23]. The vast majority of these wells (93%) have had total dissolved solids exceeding Environmental Protection Agency (EPA) standards from the time they were drilled (Figure 3). Yet, 80% of families have limited options, so they drink and/or cook with untreated well water in spite of discoloration, odor, and high levels of iron, sulfate, and total dissolved solids [23]. 

Undoubtedly, local geology contributes to poor groundwater quality, including uranium, manganese, arsenic, total dissolved solids, and hardness in aquifers within the Bighorn and Little Bighorn River watersheds [33]. However, uranium in the Bighorn River valley groundwater could be due in part to past uranium mining in the river’s headwaters as well as the extensive agricultural application of phosphate-containing fertilizer (contaminated with uranium) [23,33]. Arsenical pesticides continue to be used in cattle ranching and other non-crop applications, and we are investigating those contributions to arsenic contamination of groundwater. Elevated nitrate levels in well water are primarily found in the Bighorn River valley, where there is the most irrigated agriculture (and hence, presumably the greatest fertilizer use) [23].

Additionally, the Little Bighorn River has extremely high levels of fecal contamination, especially during spring runoff [34,35,51]. In spring and late summer, the water typically becomes unsafe even for swimming. Therefore, the aim of this study was to understand Crow community members’ perceptions of local water quality, water needs, experience with water insecurity, and actions taken to access safe water. The local water sources addressed here include not only home wells but also rivers and springs, which continue to be vitally important for traditional cultural activities.

## 3. Materials and Methods

### 3.1. Participants and Data Collection

We used purposive sampling to ensure we had a broad range of perspectives representing both genders and the six districts across the Reservation. Members of our team along with other tribal members of the CEHSC developed six open-ended interview questions to gain deeper insight into rural residents’ water access, costs of obtaining water, recreational and ceremonial activities related to water, health effects related to water exposure, actions taken to access safe water, changes in water uses over their lifetime, as well as a question about a recent flood the community had experienced. (The interview questions are available in the Supplementary Materials file.) Four Crow tribal members trained in qualitative interviewing conducted the interviews with 30 adult Crow tribal members which were audio recorded and transcribed. Interviews lasted less than an hour. Interviewees provided informed consent and received a gift card for their time. This study protocol posed minimal risk to participants and was granted exempt status by the Montana State University Institutional Review Board.

### 3.2. Qualitative Data Analysis

Our analysis team included six CEHSC members and an academic partner. The team consisted of five tribal members and the sixth member married into the tribe. We used inductive thematic analysis, where our analysis was grounded in the data [52]. First, the team members read through the interviews to become familiar with the data. Next, each member independently identified and labeled codes in the data. Four of us met to compare codes and develop themes. A third meeting was held with the full steering committee and a final list of themes was refined. The first and second author then reviewed the themes and went back to the transcripts to ensure the themes fit with the coded data. Discrepancies were reviewed and resolved through in-depth discussion. These themes were further discussed and refined by the full analysis team and other members of the CEHSC.

Through the interviews, participants shared their stories experiencing water insecurity. Apsáalooke people have great respect for the story and the storyteller. The team, which included community members, used a qualitative analytic process that consisted of many in-depth discussions regarding the interviews and study findings [53,54]. This approach respected Apsáalooke culture and the individual stories of the participants. CEHSC members played a key role verifying the accuracy of transcriptions and the interpretations of the data.

## 4. Results

Almost all of the participants had home wells, and about half of them reported that their tap water was drinkable (Table 1). However, at least half of this group still hauled water to fill their home cistern and/or bought bottled water by the case once or twice a week. Participants traveled an average of 84 miles round trip to haul water. The data in Table 1 came from questions asking participants how they obtained water for household use. Due to the open-ended nature of the interview, not everyone provided data related to hauling water. For example, people who reported that their water source was city water did not haul water and some people with wells considered their water safe to drink. 

We also identified eight themes which illustrate the experience of water insecurity in the Apsáalooke community. A central theme that emerged from our analysis was the sacredness of water and the special relationship with water that is essential to the traditional Apsáalooke way of life. The other themes we identified were related to the changes that occurred over the lifetimes of the participants and how they dealt with those changes. Solutions are hard to find, and resources are limited. Table 2 illustrates the categories resulting from the thematic analysis, along with brief descriptions for each. 

### 4.1. Water is Sacred

The sacredness of water to the Apsáalooke emerged as an underlying, central theme across the interviews. While water is of central importance to all living beings, many Apsáalooke participants explained how their spiritual connection to water extends beyond daily household uses such as drinking and bathing. Water is a central element in many of their spiritual practices including the Sweat Lodge, Sun Dance, and Native American Church ceremonies. 

For Apsáalooke people, water is imbued with a spiritual power—a living force with its own energy. Participants shared their personal experiences of growing up, living near the river and all the ways they were taught by their elders to use, respect and value water.

*Well, in the spring, when the water has flooded and it starts going down, we feed the water [to protect our children]. Our kids are safe, they play, our kids, they play at the river all day. And then we would have the Sundance, and then we give water to people in mourning, and we have a lot of uses for water. We’re always praying over water […] we want to pray over water and then give it to those [ill] people, so they feel better…*

Some participants talked about central role the river had played in their childhood experience.

*I grew up at the river and I was always there. The Little Bighorn River is right there, a few feet away from my house […] We were always playing at the river. And then in the winter when it freezes over, we skate there too […] We always said we were raised by the river, the Little Bighorn River right there.*

**Figure 4:**
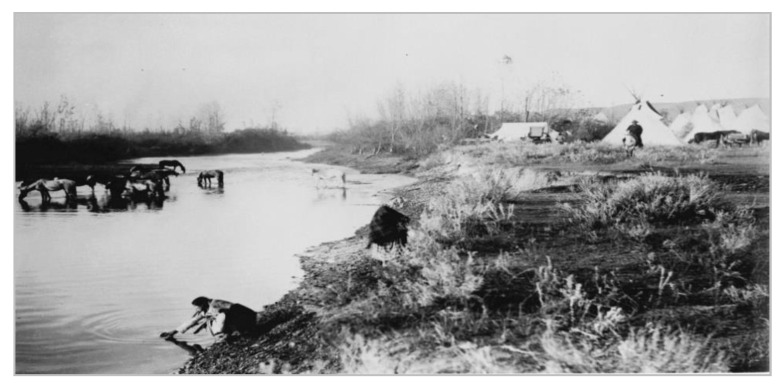
A Crow woman collecting water from the Little Bighorn River, Crow Agency, MT. This was the main water source for the Crow people before the 1960s. (Photo used with permission from The Crow Indians Photographic College Archives, Crow Agency, Montana. Original at American Anthropological Archives, Washington, DC, USA).

**Figure 5:**
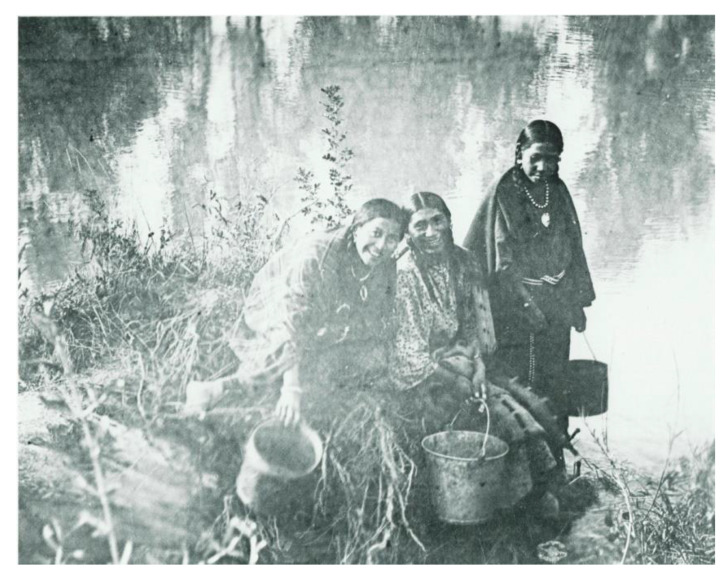
Going after Water—left to right, Mrs. Iron Horse, unidentified woman, and Lucy Driftwood, gathering water from the Little Bighorn River, ca. 1910. (Photo used with permission from The Crow Indians Photographic College Archives, Crow Agency, Montana. Original at American Anthropological Archives, Washington, DC, USA).

Figure 4 and Figure 5 are photographs taken before indoor plumbing was common in households on the reservation. They show Crow women collecting water from the river. The first photograph shows a Crow encampment along the river and the second includes women collecting water for their family to use. 

### 4.2. Causes of Change

Apsáalooke people are deeply connected to traditional and current homelands and waterways—a connection that is passed through generations. However, colonization has impacted the health of our homelands and waterways, thereby increasing water insecurity caused by the contamination of local river and streams. Participants voiced their concerns regarding increases in population and intensification of agriculture which have caused river quality to worsen. For example, this person explained how life has changed and agriculture now pollutes and depletes local water sources.

*Everything has changed now, with different people moving in. I mean when the old ranchers used to have this place, they never sprayed their crops, not like they do now. And of course, they use irrigation through the year, and I do not know whether it is the chemicals or what that is making the water worse here.*

As river water quality worsened, people lost this source of water for domestic, recreational, and ceremonial uses. Participants described how their use of river water had phased out as they got older with the implementation of modern plumbing. Others talked about how their indoor plumbing has deteriorated over time contributing to water insecurity within their household.

### 4.3. Health Risks associated with Water Quality Changes 

Water insecurity is impacted by the perceived and actual health risks associated with poor water quality. Participants expressed concern regarding microbial contamination. As one participant remarked, “My concerns are the people getting sick from the water. I do know that we have *H. pylori, E. coli, Cryptosporidium* and so my concern is we have a lot of people that do use the river”.

A few other participants also voiced their concern regarding the potential contribution of unsafe water to the development of cancer and diabetes.

*We used to fetch water right over here, me and my brothers would come home with pails and haul water back for drinking. That’s what we lived on, then when we built the big house, we had running water and my dad always drank it, but I always thought he maybe died from that because he died of cancer. It could be the hard water because the taps are orange colored and rust colored. We drank water from the river, and he drank right off the spout and he always thought it was good water, but he died of pancreatic cancer and I always wondered if it was connected or related.*

However, there were also several participants who perceived their water as safe for consumption.

*I can’t think of anybody that got sick from the water here, from the river or from the well water that we drink. My mom’s house that I grew up in was also well water, and that one is rusty. It’s really hard water, and I grew up drinking it and we always drank it and our babies drank it. Um, we mix their formulas with it, and nobody ever got sick that I can remember.*

This participant also perceived their water source as safe, and still drank it during ceremonies.

*Horses and the cows cross over it [river], but they don’t stay in the water, and there’s rocks, a lot of rocks, and there’s the water’s running all the time, so it’s pretty clean. But we drink that river during the [sweat lodges] or [Native American Church] meetings. We don’t get sick. And it’s pretty clean around where I live. But upstream, I don’t know. Must be ok because we never got sick.*

### 4.4. Resulting Changes in Water Use (Sense of Loss)

While the above participant still drinks their water source during ceremonies, most participants described a sense of loss regarding their relationship with the river. Many participants explained how water quality has changed during their lifetime and through the generations. Participants described how they no longer fish, swim in or drink from the river—because of worsening water quality.

*As I become more aware of what’s in that water, I’ve really curtailed my use of it. You know, I was thinking about it this morning, all of my life, from the time I was a little kid, that river played an important part of our life. Every bit of the fish that we caught out of there we ate. It wasn’t like we were looking for a large fish or a trophy fish or a specific type a fish. All that fish came out, my dad would take it home and my mom would cook it, and there would be a plate of mixed fish on the table…That was our food. And so, now I don’t fish anymore […] And that was something I enjoyed doing […] and I think I relayed that story to you where I got a number of catfish one time, and every one of them had sores on their body. And I had never, ever seen that in my life. And that told me right there, something was going on with that river that I had never seen. And so, you know that was a big deterrent to me.*

In addition, this participant described the switch to well water from river water.

*When I lived down there […] when I was a teenager, always they would get the drinking water right out of the river. And that was on the counter there, a bucket with a dipper, [they] would open that hole up in the ice and dip water out of there, and that was just the drinking water […] Nobody gave it a second thought […] You know. But I know that wouldn’t happen now. They preferred the river water over the well water, because the well water had that funny taste to it.*

### 4.5. Water Insecurity 

Several participants talked about the burden of having to obtain enough safe water for their household use, as well as having the physical strength and tools needed to haul large amounts of safe water. One participant described the cost of buying and hauling water in this way:

*We have to pre-pay before they deliver the water. We mostly use the water from the cistern to shower, washing dishes, and flushing the toilet. We have two five-gallon water jugs on reserve just in case they don’t deliver the water right away. For our drinking water, I buy the bottled water. I buy about four of the 24 in a case. It lasts about a month. It costs about $20. For cooking, I have a water cooler that contains a five-gallon jug [...] That costs about $5. If I add it all up in the month $215 a month.*

Participants also discussed the effects of hard water build up on the pipes and everything else, for instance:

*It’s really bad […] I’d like to have some something done for us. Get some water so we can drink it from the house. And, I don’t have to buy water all the time […] Everything’s just bad at the house. That water. Can’t even wash, can’t even do anything with it.*

Another participant further described hardship, “The quality of that well water is hard water […] and you have to use one of [those] water softeners and all that to get it to work right”.

### 4.6. Dealing with Change

Participants were asked what they had done to make sure the water they used was safer and cleaner. Participants described having their home well water tested, and then taking next steps to clean the water.

*Little Big Horn College’s program came and did testing on it. There were bacteria in there. I had to have [the well] shock chlorinated and there were still bacteria in there and that is why I had to turn to buy bottled water for drinking and cooking with. I don’t know how it is today. I probably still have to shock chlorinate it to get rid of that. I did get sick from drinking my water. When we first moved in, the house water had a lot of iron in it. I had to put in a water softener, and it helped, and it was ok. Until maybe within the last three years we quit drinking it after we found out I had ulcers from the bacteria in there.*

### 4.7. Solutions are Hard to Find 

As people answered the question about what they had done to make their water safer, many participants also talked about the many impediments to taking action to make their water safer. Several participants discussed feelings of frustration for where to turn for help with their water issues. “It’s really bad and I have nobody to help us, to fix the pipes and everything…” 

And there was further frustration regarding the responsibility for fixing well-water issues and ensuring safe drinking water. One participant described it in this way:

*And they only drill until they hit first water and that’s as far as they go. We were told when we had our well done here that they were going to dig, do four different tests, and they would tell us what the results were. And they did samples of how far down it was, and they took samples of soil, and the water and didn’t even take them. They didn’t test them, I kept complaining kept complaining and so far, nothing has been done. And like I said you know it’s something they do for us, but yet at the same time they don’t do it right…*

Some participants talked about having a limited understanding of why their home well water was unsafe to drink. Other participants talked about how they have trouble locating the resources needed to address their poor water quality and what they use to understand if their well water was safe or unsafe to drink and use.

*I know in Lodge Grass they always tested it, and my sister worked there so I always knew that it was safe, but other than that I don’t know how it is now. And here in Hardin, they never send any letters saying that they tested the water or anything. I assume you know that they do. It hasn’t tasted any different.*

Another participant described it in this way:

*Well, we don’t know that for sure, we don’t know if it’s because this flood is right here in Wyola and I don’t know for some reason we were told up here, we are about seven miles towards the Little Bighorn [river], towards the mountains, and we were told we weren’t supposed to use the water. As far as I know it’s the same water that flows here in the Little Bighorn that’s in town there, they’re getting but we feel that for some reason it’s safer […] I know that we were told not to use it, so far we have been kind of taking their advice and getting all our water down in Wyola from families.*

### 4.8. Availability of Resources 

Although water quality in the Apsáalooke community is a complex issue that is not easy to solve, there are potential resources in the community. For example, the Apsáalooke Water and Wastewater Authority has been able complete over $20 million worth of renovations and repairs on Crow Agency’s public water infrastructure, backed by CEHSC water quality research and their partners at Montana State University-Bozeman [32]. This participant described the resources provided by the water quality group.

*These guys were trying to actually improve the drinking the water, then they did the study and found out the city pipes were bad. It was the water quality group here that has been testing it. They even tested the water down by our river. They tested our house water and said for the most part it was ok.*

This testing has been helpful for community members who have contaminated water, so that they may understand and take action. However, further resources are needed to continue water testing and to assist with mitigation. One participant discussed their experience with water quality testing.

*Because of our cattle in the winter they are in our pasture and I think the manure seeps into it, and they tested before and they told us not to drink it, so we buy our own water. I think they need to test it again.*

## 5. Discussion

Apsáalooke participants in our study emphasized how local surface water sources have deteriorated over time. The resulting changes have caused many tribal members to experience water insecurity. As river water quality has worsened, access to safe, clean, free water for basic household needs has been lost. While water insecurity is distressing for any community, there are added dimensions of water insecurity that make it more complex for the Apsáalooke. First and foremost, there is the deeper spiritual relationship with water that is embedded within Apsáalooke cultural practices. Second, water insecurity is impacted by lack of financial resources and inadequate community capacity to address plumbing and water resource management issues. Finally, there are the complex tribal jurisdictional issues that make mitigating water contamination issues difficult [39].

For participants in this study, cultural traditions were also heavily impacted by worsening water quality of rivers and springs. Using water from these natural sources has always been an essential component of many Apsáalooke ceremonies. Although most participants reported they no longer drink untreated river water for domestic use, this potentially contaminated water may still be consumed during ceremonies. The Apsáalooke are not oblivious to the health risks associated with potential spring and river water contamination. However, cultural practices and a relationship to water are what make us Apsáalooke. The potential impacts of consuming contaminated water are weighed against something more pernicious to health—losing that relationship, which is central to who we are as Apsáalooke. Similarly, the Swinomish tribe of the Northwest, have had their waters and hence their traditional seafoods contaminated. For them, seafood is central to their identity and to ask them to reduce their consumption of local seafood is “detrimental their concept of health [25]”.

Our findings confirm recent research on water security within other Indigenous Nations [26,27,28,29,55]. Our study also confirmed that the impacts of water insecurity are not merely conceptualized in material physical terms but are also understood in cultural and spiritual terms [27,28]. For many Indigenous people, water is inherently connected to our health and well-being, requiring that research on water insecurity also consider cultural values connected to water. Recently, researchers from Canada used in-depth interviews with tribal members to investigate the meaning of “water security” from an Indigenous perspective [28]. In their exploration they described a relationship where water provides for the people and in turn the people provide for it (reciprocity). These authors outlined two perspectives: One represents material Western science and is the focus of most of the literature on water security [4], and the other, Indigenous perspective, which includes spirituality and tradition and is often missing from the literature on water insecurity. These two views need not be antagonistic and may in fact both be necessary when considering water insecurity within Indigenous communities [28].

In addition to cultural and spiritual impacts of water insecurity, participants also described the material hardships they faced due to contaminated water. The poverty rate for the Crow Reservation was 31.5% in 2015, compared to 10.5% for the US general population [56,57]. Limited financial and technical resources make the challenges associated with water insecurity even more daunting [29]. For those with home wells, mitigating unsafe well water is complicated and expensive. Because of the complex groundwater contamination issues in the area, most people with bad water would need a water softener, an iron removal unit, and a reverse osmosis unit. Even if families could afford this initial investment, these units incur additional monthly costs for chemicals and maintenance. According to Indian Health Service data, 93% of new wells on the Crow Reservation (*n* ~ 500) exceeded the EPA standard for total dissolved solids [23]. Hence, they are so high in minerals that a simple filtration system is inadequate, and the generally hard and/or alkaline water will continue to deteriorate the plumbing. Although there is limited assistance from the Indian Health Service, the opportunity to have a well drilled or a cistern installed is offered to tribal members only once in their lifetime; after the installation, maintenance and repairs are up to the homeowner. 

In addition, mitigation is made more complex because participants felt they lacked access to people and resources to provide current information about water quality concerns. Most of the participants in this study were on home wells or cisterns. Although municipal water sources are monitored for safety, the federal government does not generally regulate the quality of home well water. Furthermore, some of the contaminants common in Apsáalooke groundwater such as arsenic, uranium, and nitrate may be colorless and odorless, yet, may still be harmful. These contaminants contribute to risks for chronic conditions including diabetes and cancer; people may not realize that water contamination increases their risks associated with these diseases [23,39].

Like other studies promoting well stewardship, members of our team have found that while well water testing is useful, community members would like more interactive educational methods in addition to print reports [58]. We have found that the best method for providing education regarding water-quality within the Apsáalooke community is person-to-person [23]. Through these conversations we can make personal connections with community members. They often take place in people’s homes and when we see them out in public at the grocery store or post office. However, with the recent Covid-19 outbreak, in-person communication has been made more difficult. We are not able to conduct home-visits in the same manner and even when we meet community members out in public places, social distancing takes away some of the personal connection. There is a loss of privacy when we try to talk about personal household water issues out in public—and discuss while 6-feet apart. 

Additionally, there are no community-based opportunities for homeowners to learn how to take care of their wells, cisterns, septic tanks, or indoor plumbing. In some rural communities, families have had wells over generations, and thus have learned how to manage their wells and plumbing. However, the Apsáalooke relied on their rivers and springs for generations, it was only more recently, in the 1960s, when wells and indoor plumbing were installed in homes in the more rural areas of the reservation. Thus, most families lack generational knowledge regarding well stewardship. 

**Figure 6:**
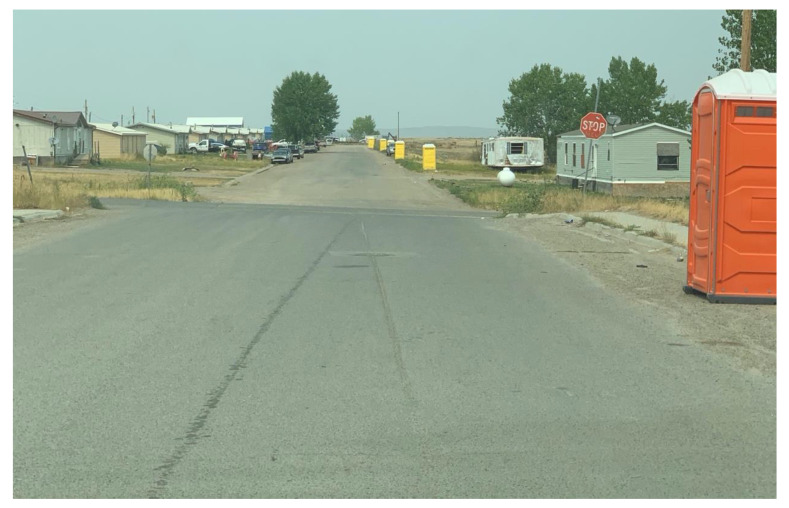
Portable toilets on the street for residents to use while water restrictions are in place for the town of Crow Agency, MT August 2020. Photo credit: Christine Martin.

Further, there is no effective public health oversight of well and septic system location planning, installation or maintenance. In our work throughout the Reservation, we frequently find septic systems installed less than 100′ from home wells (as required in Montana), old homes widely believed to be piping their raw sewage straight into the river, and failed septic systems leaking sewage to the ground surface. Public health oversight and regulatory functions governing onsite wastewater systems are simply lacking. And, even if enforcement of public health regulations was attempted, many if not most families would not be able to afford the costs of bringing failed (or absent) septic systems into compliance. A similar lack of adequate planning has applied to Tribal housing, with homes built in locations that could not be effectively served by existing public water and wastewater services, resulting in homes without running water or wastewater collection. More recently, residents of Crow agency experienced severe water insecurity when water restrictions were put in place due low pressure in the towns water tanks. Figure 6 is a photograph of the portable toilets the BIA provide to alleviate the stress caused by inadequate water supply. 

Another seemingly insurmountable barrier is the mixed jurisdiction issue [39,59]. The tribe has no control over sources of water contamination from non-tribal agricultural operations, such as fertilizer, pesticides, and manure. People have watched as agricultural activities have intensified over the years and river water quality has declined accordingly. Several participants expressed feelings of powerless to do anything about it, to the point that a couple people have commented, “I wish you hadn’t even told me [about the water contamination issues]”. While Tribes do have the option to apply for “Treatment as a State” status to set and try to enforce their own water quality standards, the administrative, scientific, and political capacities required to achieve this status are formidable. This jurisdictional issue is a unique aspect of water insecurity faced by tribal nations. 

This study highlights the significance of cultural and spiritual impacts of water insecurity that must be included in assessments of water insecurity and interventions to address water insecurity in Indigenous communities. Although this study has many strengths, it may not be generalizable to other tribal communities; the purpose was to understand water insecurity among the Apsáalooke community and provide an overview of the burdens associated with water insecurity faced by our tribal members. Our intention was to have a sample of adult participants representing the seven districts across the 2.1 million acres of the rural reservation. We did not assess the variation in socioeconomic status across participants and realize that SES impacts the relative hardships associated water insecurity. However, overall, many Apsáalooke face greater economic hardship than the average Montana or US citizen, with the median per capita income for Crow community residents (Lodge Grass, Crow Agency, Wyola, and Pryor) being approximately half the Montana average [56,57]. Additional research is needed to further develop and evaluate local, affordable mitigation strategies to ensure water security. 

More recent challenges are only adding to these burdens, including climate change and the Covid-19 pandemic. Climate change adaptation and mitigation to reduce impacts to local water and health is also needed, as the Tribe is already experiencing some of those effects [32,36,60]. Over the past decades, our tribal community has noticed declining winter snowfall, milder winters and hotter summers, more frequent spring floods, negative impacts on subsistence species, and extreme changes in our weather patterns, making our seasons unpredictable and putting strain on our natural resources and community health. Some community members are aware of the impacts from climate change, and can compare to previous years, while others are too young to understand the difference in the climate. In addition, the challenges of water insecurity have only intensified amidst the recent Covid-19 pandemic. First, there are the usual costs associated with driving up to 200 miles round trip each week to purchase enough drinking water for the household; however, this hardship is now coupled with the additional risk of exposure to the virus while shopping. Furthermore, water insecurity complicates basic household sanitation—an important step in preventing spread of the virus during a pandemic.

## 6. Conclusions

The Crow Environmental Health Steering Committee has worked since 2004 to address material challenges including providing water testing, education, and water coolers. We are currently developing other mitigation efforts including securing a septic tank pumping truck, installing cisterns, harvesting rainwater, and educating local health providers about the health effects associated with water contamination. We are addressing water insecurity holistically through our efforts to educate local youth about water quality science, cultural significance of local water sources, and Apsáalooke values respecting rivers and springs [61,62,63].

Although a few studies have examined water insecurity in Indigenous Canadian groups [26,27,28,55], our study is one of the few to examine the topic of water insecurity within an Indigenous community in the US. This study broadens our understanding of the impacts of water insecurity among Apsáalooke community members, including the importance of taking spiritual and cultural impacts of water insecurity on health into account when working to elucidate how water insecurity impacts health [41]. The challenges associated with understanding and addressing water insecurity are complex and difficult to overcome particularly for Native American who have a special relationship with water and who must grapple with economic hardships and complex or inadequate jurisdiction. Water insecurity is a growing global problem and more attention and efforts are needed to find appropriate and affordable solutions. 

## Figures and Tables

**Table 1:** Household water sources and associated water hauling costs per participant.

Participant	Water Source	Drinkable	Amount of Water	How Often?	Cost of Water per Trip	Miles Roundtrip	Total Cost per Month
1	Well	Yes	2.5 cases	Weekly	15.00	200	$520.00
2	Cistern	*	1000 gal	Monthly	25.00	28	$41.20
3	*	*	5–10 gal	Weekly	12.00	80	$232.00
4	Cistern	*	250 gal	Weekly	50.00	*	**
5	Well	Yes	*	*	*	*	**
6	Well	Yes	*	*	30.00	140	**
7	Well	Yes	*	*	*	*	**
8	Well	No	5 gal	Weekly	20.00	80	$264.00
9	Well	Yes	*	*	*	*	**
10	Well	Yes	10 gal	Weekly	6.00	*	**
11	Well	No	1 case	Weekly	*	80	**
12	Well	Yes	10 gal	Weekly	6.00	*	**
13	Well	Yes	*	*	*	*	**
14	Well	Yes	*	*	*	*	**
15	Well	Yes	*	*	*	*	**
16	Well	No	4 cases	Weekly	*	*	**
17	Well	No	Haul	Weekly	60.00	*	**
18	Cistern	Yes	Haul	Weekly	32.00	24	$183.20
19	City Water	*	*	*	*	*	**
20	*	*	*	*	*	*	**
21	Well	Yes	*	*	*	*	**
22	City Water	*	*	*	*	*	**
23	City Water	*	*	*	*	*	**
24	Cistern	Yes	*	*	*	*	**
25	Well	Yes	Haul	Monthly	115.00	*	**
26	Well	No	*	Monthly	50.00	80	$96.00
27	City Water	*	*	*	*	*	**
28	Well	No	Haul	Weekly	*	80	**
29	Well	Yes	*	*	*	*	**
30	Well	No	20 gal	Weekly	100.00	50	$515.00

* This information was gathered from open-ended qualitative interviews and some participants were not asked or did not answer the specific question to elicit information. ** Unable to calculate based on available data.

**Table 2:** Themes and their descriptions resulting from qualitative analysis.

Theme	Brief Description of Theme
Water is Sacred	Traditional relationship and respect that Apsáalooke have with river and springs.
Causes of Change	Perception of causes of changes to water insecurity. For example, increases in population and agricultural use of local water has caused river water quality to worsen; the introduction of modern plumbing has caused Apsáalooke people to obtain their drinking water from the tap instead of going to the river or spring which changes the special relationship between people and their water sources.
Health Risks associated with Water Quality Changes	Perception of health risks associated with local water quality changes which impact water security.
Resulting Changes in Water Use (Sense of Loss)	Changes in the way Apsáalooke use water. For example, water for domestic and ceremonial uses coming from tap water instead of river water; not fishing in local rivers anymore.
Water Insecurity	Burdens associated with having to obtain enough safe water for household use.
Dealing with Water Insecurity	Actions or activities undertaken by Apsáalooke to address water insecurity.
Solutions are Hard to Find	Challenges people faced when working to address water insecurity.
Availability of Resources	Availability of resources in the community to address water insecurity.

## Data Availability

The data presented in this study are available on request from the corresponding author. The data are not publicly available due to ethical reasons.

## Supplementary Materials

The following are available online at https://www.mdpi.com/1660-4601/18/2/582/s1, Interview questions for “Water—A Resource for Health”.
