# Supplementary material for: Our Relationship to Water and Experience of Water Insecurity among Apsáalooke (Crow Indian) People, Montana
Source: Int J Environ Res Public Health. 2021 Jan 12;18(2):582. doi: 10.3390/ijerph18020582 (PMC7827827; doi:10.3390/ijerph18020582)
Supplement: Supplementary file 1 [file ijerph-18-00582-s001.pdf]

International Journal of *Environmental Research and Public Health*

MDPI

Article

Supplementary Materials: Our Relationship to Water and Experience of Water Insecurity among Apsáalooke (Crow Indian) People, Montana

Christine Martin [1,2,3,*], Vanessa W. Simonds [1,4,*], Sara L. Young [1,3,5], John Doyle [1,2,3,6,7], Myra Lefthand [1,3] and Margaret J. Eggers [3,7]

[1] Crow Tribe of Indians, Crow Agency, MT 59022, USA; saralyoung@hotmail.com (S.L.Y.); doylej@lbhc.edu (J.D.); myrajlefthand@gmail.com (M.L.)
[2] Crow Water Quality Project, Little Big Horn College, Crow Agency, MT 59022, USA
[3] Crow Environmental Health Steering Committee, Little Big Horn College, Crow Agency, MT 59022, USA; mari.eggers@montana.edu
[4] Department of Health and Human Development, Montana State University, Bozeman, MT 59717, USA
[5] Center for Health Equity Research, Northern Arizona University, Flagstaff, AZ 86001, USA
[6] National Environmental Justice Advisory Council, Environmental Protection Agency, Washington, DC 20460, USA
[7] Department of Microbiology & Immunology, Montana State University, Bozeman, MT 59717, USA
* Correspondence: martinc@lbhc.edu (C.M.); vanessa.simonds@montana.edu (V.W.S.)

Citation: Martin, C.; Simonds, V.W.; Young, S.L.; Doyle, J.; Lefthand, M.J. ; Eggers, M. Our Relationship to Water and Experience of Water Insecurity among Apsáalooke (Crow Indian) People, Montana. *Int. J. Environ. Res. Public Health* **2021**, *18*, 582. https://doi.org/10.3390/ijerph18020582

Received: 29 September 2020
Accepted: 6 January 2021
Published: 12 January 2021

Publisher's Note: MDPI stays neutral with regard to jurisdictional claims in published maps and institutional affiliations.

Copyright: © 2021 by the authors. Licensee MDPI, Basel, Switzerland. This article is an open access article distributed under the terms and conditions of the Creative Commons Attribution (CC BY) license (http://creativecommons.org/licenses/by/4.0/).

Interview questions for "Water—A Resource for Health"

1. Introduction

The purpose of these survey questions is to find out how people are using water, including rivers, streams, springs, lakes, and water for home use. If we can find out what the common problems are with water, we can look for solutions that will help people. This project is a joint effort of Messengers for Health and the Crow Environmental Health Steering Committee, which includes representatives of the Crow Tribe, the Apsáalooke Water and Wastewater Authority, the Crow/Northern Cheyenne Indian Health Service hospital, the 107 Committee and Little Big Horn College.

What you say is your voice, we want to make sure we get everything you say and get it right. We would like to tape record our conversation, to make sure we get it right. Is that alright with you?

2. Questions

1 Where do you get your water?

[If they haul water, follow up with: How much time does it take you to go get water? How many times a week/month do you go? How much does the water cost you? What type of transportation do you use?]

2 What are some cultural and recreational activities that you participate in involving rivers, streams, springs, and lakes?

3 Over your lifetime, have you changed how you use rivers/streams/springs/well water?

[If there have been changes, follow up with: How have things changed? What contributed to causing those changes?]

(We would like to know if pollution has been a contributing factor, but don't want to ask a leading question.)

4 Have you or any members of your family ever gotten sick from the water? (home water, municipal water, or river or spring water?)

5 What have you done to make sure the water you and your family use is safer and cleaner?

6 Did this spring's flooding affect the availability or safety of your drinking water? (If so, how?)

Thank you for your time and participation to help us improve the health of our community.
